# NPRL2 is required for proliferation of oncogenic Ras-transformed bronchial epithelial cells

**DOI:** 10.1186/s13008-024-00126-w

**Published:** 2024-06-24

**Authors:** Jing-Yuan Chuang, Hsiao-Hui Kuo, Pei-Han Wang, Chih-Jou Su, Ling-Huei Yih

**Affiliations:** 1 Department of Medical Laboratory Science and Biotechnology, China Medical University, Taichung, Taiwan; 2 Institute of Cellular and Organismic Biology, Academia Sinica, Taipei, 115 Taiwan

**Keywords:** NPRL2, mTORC1, Oncogenic HRas, Bronchial epithelial cells, Cell proliferation

## Abstract

Nitrogen permease regulator-like 2 (NPRL2/TUSC4) is known to exert both tumor-suppressing and oncogenic effects in different types of cancers, suggesting that its actions are context dependent. Here, we delineated the molecular and functional effects of NPRL2 in malignantly transformed bronchial epithelial cells. To do so, we depleted NPRL2 in oncogenic HRas-transduced and malignantly transformed human bronchial epithelial (BEAS2B), Ras-AI-T2 cells. Intriguingly, depletion of NPRL2 in these cells induced activation of mTORC1 downstream signaling, inhibited autophagy, and impaired Ras-AI-T2 cell proliferation both in vitro and in vivo. These results suggest that NPRL2 is required for oncogenic HRas-induced cell transformation. Depletion of NPRL2 increased levels of the DNA damage marker γH2AX, the cell cycle inhibitors p21 and p27, and the apoptosis marker cleaved-PARP. These NPRL2-depleted cells first accumulated at G1 and G2, and later exhibited signs of mitotic catastrophe, which implied that NPRL2 depletion may be detrimental to oncogenic HRas-transformed cells. Additionally, NPRL2 depletion reduced heat shock factor 1/heat shock element- and NRF2/antioxidant response element-directed luciferase reporter activities in Ras-AI-T2 cells, indicating that NPRL2 depletion led to the suppression of two key cytoprotective processes in oncogenic HRas-transformed cells. Overall, our data suggest that oncogenic HRas-transduced and malignantly transformed cells may depend on NPRL2 for survival and proliferation, and depletion of NPRL2 also induces a stressed state in these cells.

## Introduction

Nitrogen permease regulator-like 2 (NPRL2, also known as tumor-suppressor candidate 4; TUSC4) is a requisite subunit of the conserved GAP activity toward Rags 1 (GATOR1) complex that inhibits mechanistic target of rapamycin complex 1 (mTORC1) in response to amino acid insufficiency [1]. mTORC1 acts as a serine/threonine kinase to integrate upstream nutrient and growth factor signals with downstream transcriptional and translational apparatuses that control cellular biomass accumulation and metabolism. As such, mTORC1 plays major regulatory roles in fundamental cellular processes including energy utilization, protein synthesis, autophagy, cell growth and proliferation [2]. In light of its myriad functions, the mTOR pathway is widely considered to be a central regulator of cellular and physiological homeostasis, and dysregulation of mTOR signaling has been implicated in many cancers [3]. Since modulating the activity of TORC1 with GATOR1 can rapidly fine-tune the cellular metabolic state in response to environmental cues [4], these factors are able to coordinate cell growth/proliferation with environmental/physiological status.

Much work on NPRL2 has been conducted using Drosophila. In this model organism, NPRL2 is required for viability, and the lethality associated with the NPRL2-null mutation is due to the hyperactivation of TORC1 [5]. Furthermore, NPRL2 RNAi or a null mutation that fails to downregulate TORC1 in response to amino-acid limitation will trigger apoptosis in fly egg chambers [5, 6], implying that NPRL2 protects the cells during amino-acid starvation. However, in the escaper adults of NPRL2-null mutants, multiple cell types exhibit significantly increased sizes, suggesting that NPRL2 also constrains cell growth in some circumstances [5]. Along these lines, a loss-of-function NPRL2 mutation in flies was shown to increase TORC1 activity and induce a high rate of intestinal stem cell proliferation [7]. Genetic studies in yeast supported this idea, as mutants lacking NPRL2 exhibit unchecked growth under specific nutrient limitations [8]. Taken together, these studies reveal that NPRL2 inhibition of TORC1 may have different effects on cell proliferation in different cellular contexts and at different stages of cell development.

Loss of a genomic locus (3p21.3) containing NPRL2 and decreased NPRL2 expression are observed in many cancer cell lines and tumor tissues [9–11], suggesting that the protein may have tumor suppressive functions. However, the expression of NPRL2 is slightly and significantly increased in lung cancer tissues compared to macroscopically unchanged lung tissues surrounding the primary lesion [12], making it unclear whether NPRL2 consistently acts as a tumor suppressor. In addition, high expression of NPRL2 has been linked to poor prognosis in patients with prostate cancer [13], and NPRL2 expression level is known to be upregulated in prostate cancer cell lines [14]. Importantly, NPRL2 overexpression was shown to promote proliferation and docetaxel chemoresistance of prostate cancer cells through a mechanism that involves inhibition of the mTOR pathway [15, 16]. Thus, the effects of NPRL2 expression on mTOR may be especially important for cell survival and cell proliferation in the context of various cancers.

mTORC1 integrates a variety of extracellular and intracellular signals to drive metabolic pathways that facilitate cell growth and proliferation, and aberrant hyperactivation of mTOR signaling has been observed in many solid tumors [17, 18]. For this reason, selective mTOR inhibitors are under evaluation in clinical trials on different cancers [19]. However, mTOR hyperactivation may also disrupt cellular homeostasis and induce cell death, likely by mechanisms involving increased ROS and oxidative stress [20, 21]. Thus, mTOR activity levels must be titrated in cancer cells such that the cell can meet the high energy demands of cell growth/proliferation without causing cell death. We hypothesized that NPRL2 may play an essential role in restraining mTORC1 activity in transformed cells to allow for survival and proliferation of the transformed cells. Oncogenic Ras is known to induce hyperactivation of mTOR pathway [22, 23], and inhibition of mTOR can reverse oncogenic Ras-induced epithelial neoplasia [24]. Thus, we chose to investigate the role of NPRL2 in oncogenic HRas-transduced and malignantly transformed bronchial epithelial cells. Our results show that depletion of NPRL2 suppresses oncogenic HRas-transformed cell proliferation both in vitro and in vivo, likely by inducing cell cycle arrest and mitotic catastrophe and by suppressing cytoprotective stress responses. Therefore, NPRL2 appears to be required for survival and proliferation of the oncogenic HRas-transformed cells, and the cells may also depend on NPRL2 for transformation.

## Results

### Expression of NPRL2 is upregulated in lung tumor tissues

**Fig. 1:**
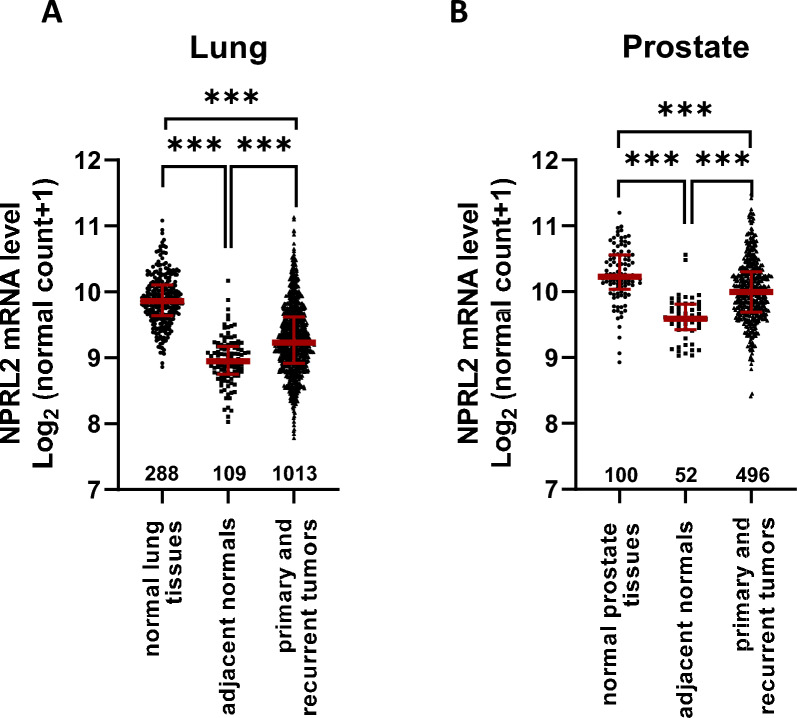
Expression of NPRL2 is upregulated in lung tumor tissues. The mRNA expression levels of NPRL2 gene were analyzed by RNA sequencing in lung (**A**) and prostate (**B**) tissues; data were retrieved from TCGA and GTEx databases using the UCSC Xena platform. Data are presented as individual points with median and interquartile ranges from (**A**) 288 normal lung tissues, 109 lung tumor adjacent normal tissues, and 1013 primary and recurrent lung tumors. **B** NPRL2 gene expression data are from 100 normal prostate tissues, 52 prostate tumor adjacent normal tissues, and 496 primary and recurrent prostate tumors. ***p < 0.001 according to Student’s *t*-test

We evaluated the mRNA expression level of NPRL2 in lung and prostate tumors using data from the The Cancer Gemone Atlas (TCGA) and Genotype-Tissue Expression (GTEx) datasets. We used the UCSC Xena platform [25] to retrieve the NPRL2 gene expression RNA-sequencing data from TCGA and GTEx. When comparing TCGA-lung tumors and GTEx-normal lung tissues, we found that NPRL2 expression was significantly lower both in lung tumors and the adjacent normal tissues than in normal lung tissues (Fig. 1A, p < 0.001), suggesting that NPRL2 may play a tumor suppressive role in lung tumor development. However, we also found that the expression level of NPRL2 was significantly higher in lung tumors than in the adjacent normal tissues (Fig. 1A, p < 0.001), suggesting NPRL2 function might be required in the malignantly transformed cells to somehow benefit their viability and/or further transformation. Similar trends of NPRL2 expression were also observed in prostate tumors and corresponding normal tissues (Fig. 1B, p < 0.001).

### Depletion of NPRL2 activates mTORC1 downstream signaling and inhibits autophagy in Ras-transformed cells

**Fig. 2:**
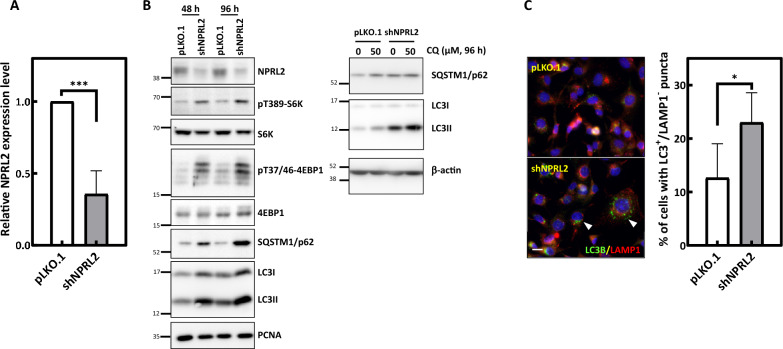
Depletion of NPRL2 activates mTORC1 downstream signaling and inhibits autophagy in oncogenic HRas-transformed cells. **A** The NPRL2 mRNA expression level in Ras-AI-T2 cells transduced with virions containing empty vector (pLKO.1) or shRNA-targeting NPRL2 (shNPRL2). Forty-eight hours after transduction (20 μL virion-containing medium/10^4^ cells), the level of NPRL2 was analyzed by qPCR. Data are the mean ± SD from three independent experiments. ***p < 0.001 according to Student’s *t*-test. **B** NPRL2 depletion activates mTORC1 and inhibits autophagy. Left, Ras-AI-T2 cells were transduced with control or NPRL2 shRNA containing virion (20 μL virion-containing medium/10^4^ cells) for 48 h or 96 h. Then, immunoblotting analyses were performed to measure the levels of NPRL2, mTORC1 downstream substrates, and autophagy markers. Right, Ras-AI-T2 cells were transduced as described above and treated with 50 μM chloroquine (CQ) for 96 h. Immunoblotting analyses were performed to measure the level of autophagy markers. **C** NPRL2 depletion increased the percentage of cells with puncta that were LC3-positive and LAMP1-negative. Left, representative images of control and NPRL2-depleted Ras-AI-T2 cells stained for LC3 (green) and LAMP1 (red). Nuclei were counterstained with DAPI (blue). Scale bar, 10 μm. Arrowheads indicate the cells with puncta stained only for LC3 and not LAMP1. Right, the percentages of cells displaying puncta stained only for LC3 but not LAMP1. Results are the mean ± SD from at least 200 cells for each condition, as determined from three experiments. *p < 0.05 according to Student’s *t*-test

We next wanted to understand the possible role NPRL2 might play in cancerous cells, particularly in those with high mTORC1 activities. Since oncogenic HRas transformation was reported to activate mTORC1 signaling [23, 26], we depleted NPRL2 in oncogenic HRas-transduced and malignantly transformed Ras-AI-T2 cells and measured mTOR downstream signaling and cell proliferation. In our experiments, the depletion efficiency of NPRL2 in Ras-AI-T2 cells was verified by qPCR (Fig. 2A) and immunoblotting (Fig. 2B, left). As previous studies reported that cells with NPRL2 depletion display increased mTORC1 downstream signaling irrespective of amino acid status [27, 28], we examined the effects of NPRL2 depletion in the presence of complete medium. Our results showed that the levels of phospho-Thr386 p70 S6 kinase (pT389-S6K) and phospho-T37/46-4EBP1 (pT37/46-4EBP1), two mTORC1 downstream signaling substrates, were significantly increased (Fig. 2B) in NPRL2-depleted Ras-AI-T2 cells compared to controls transduced with virions containing empty vector. Since activation of mTORC1 leads to inhibition of autophagy [29], the effects of NPRL2 depletion on autophagy markers were also assessed. Ras-AI-T2 cells depleted of NPRL2 had accumulation of LC3-II and SQSTM1/p62 (Fig. 2B, left). Since the simultaneous accumulation of these proteins indicates blockage of autophagy flux [30], we concluded that autophagy might be blocked in the NPRL2-depleted cells. To confirm this, the NPRL2-depelted cells were treated with chloroquine (CQ), an autophagy blocker. The results (Fig. 2B, right) showed that the control depleted cells treating with CQ accumulated elevated levels of SQSTM1/p62 and LC3-II as expected. However, the levels of SQSTM1/p62 and LC3-II in CQ-treated NPRL2-depleted cells did not further increased compared to those without CQ treatment (Fig. 2B, right). Since CQ did not further enhance NPRL2 depletion-induced p62 and LC3-II accumulation, that NPRL2 depletion may also blocked autophagy propagation. In addition, our immunofluorescence staining revealed that the number of cells with puncta stained positively for LC3 but not LAMP1 (Fig. 2C left, arrowheads) was significantly increased in the NPRL2-depleted cultures (Fig. 2C right). Since the puncta show no signal for the lysosome marker LAMP1, we can infer that NPRL2 depletion may disrupt fusion of autophagosomes with lysosomes. Overall, our results confirmed that NPRL2 is effectively depleted in Ras-AI-T2 cells and that its depletion induces mTORC1 activation and disrupts autophagy flux.

### NPRL2 depletion impedes proliferation of oncogenic HRas-transformed cells

**Fig. 3:**
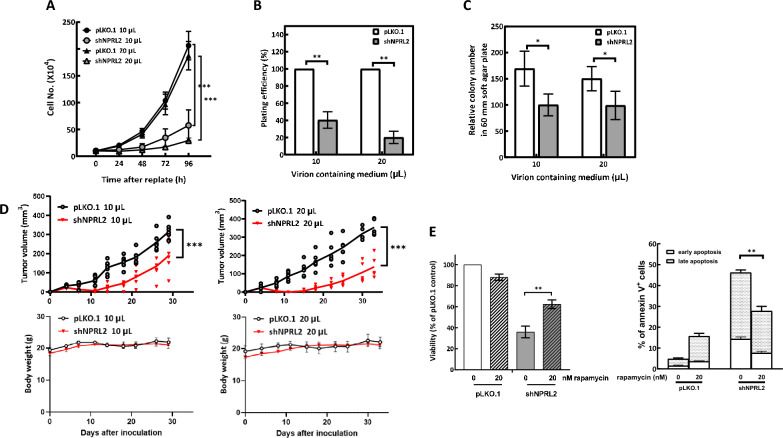
NPRL2 depletion impedes proliferation of the oncogenic HRas-transformed cells. **A** NPRL2 depletion reduces the proliferation of Ras-AI-T2 cells. Ras-AI-T2 cells were transduced with control or NPRL2-targeting shRNA-containing virions (10 or 20 μL virion-containing medium/10^4^ cells) for 48 h. Then, the cells were replated, and the viable cell numbers were monitored for 96 h at 24-h intervals with trypan blue dye exclusion assay. Results are the mean ± SD from three experiments. ***p < 0.001 by two-way ANOVA. **B**, **C** NPRL2 depletion reduced colony formation ability of Ras-AI-T2 cells. Ras-AI-T2 cells were transduced with control or NPRL2-targeting shRNA-containing virions (10 or 20 μL virion-containing medium/10^4^ cells) for 48 h. Then, the cells were subjected to colony formation (**B**) or soft agar colony formation (**C**) analyses. Results are the mean ± SD from at least three experiments. *p < 0.05 and **p < 0.01 by Student’s *t*-test. **D** NPRL2 depletion reduces the ability of Ras-AI-T2 cells to form xenografted tumors. Ras-AI-T2 cells were transduced with control or NPRL2-targeting shRNA-containing virions (10 or 20 μL virion-containing medium/10^4^ cells) for 48 h. Then, the cells were collected and used for xenograft tumor formation assays (3–4 mice in each group). Tumor volumes (upper panels) are shown for individual mice (points) and the mean for each group (line). ***p < 0.001 by two-way ANOVA. Body weights (mean ± SD) are shown in lower panels and did not differ between groups. **E** Rapamycin reduces NPRL2 depletion-induced cell death and apoptosis. Left, Ras-AI-T2 cells were transduced with control or NPRL2-targeting shRNA-containing virions (20 μL virion-containing medium/10^4^ cells) for 48 h. Then, the cells were replated in the absence or presence of 20 nM rapamycin for 72 h, and the viable cell numbers were measured with trypan blue dye exclusion assay. Alternatively, the cells were subjected to flow cytometry analysis of annexin V-positive cells (right). Results are the mean ± SD from three experiments. **p < 0.01 by Student’s *t*-test

Next, we tracked the cell proliferation rate of Ras-AI-T2 cells depleted of NPRL2 in vitro and in vivo. As shown in Fig. 3A, the proliferation rate of cells transduced with virions containing NPRL2-targeting shRNAs (10 or 20 μL virus-containing medium/10^4^ cells) was considerably less than the rate of proliferation for cells transduced with empty control virions. In addition, the colony forming abilities of NPRL2-depleted cells both in solid plates (Fig. 3B) and in soft agar (Fig. 3C) were also significantly decreased compared to cells transduced with empty control virions. We then evaluated cell proliferation in vivo by generating xenografted tumors in immunodeficient mice. In this experiment, proliferation was also significantly reduced by depleting NPRL2 in Ras-AI-T2 cells (Fig. 3D). Additionally, our results shown in Fig. 3E indicated that the NPRL2 depletion-induced inhibition of cell proliferation (Fig. 3E, left) and induction of apoptosis (Fig. 3E, right) could be considerably reduced by rapamycin, a mTORC1 inhibitor. This result implies that NPRL2 depletion-induced mTORC1 activation might cause cell death in Ras-transformed cells. Taken together, these experimental results indicated that NPRL2 depletion impairs proliferation of oncogenic Ras-transformed bronchial epithelial Ras-AI-T2 cells both in vitro and in vivo. Thus, the oncogenic Ras-transformed cells may depend on NPRL2 to restrain mTORC1 for proliferation and malignant transformation*.*

### NPRL2 depletion induces cell cycle arrest and mitotic catastrophe

**Fig. 4:**
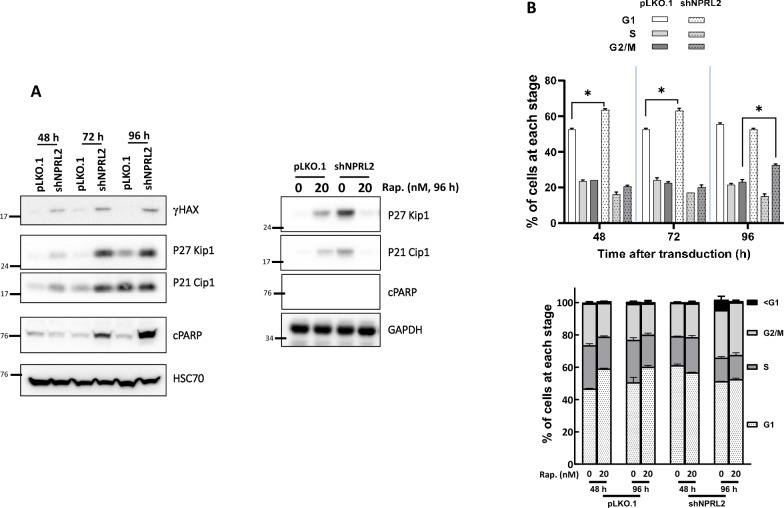
NPRL2 depletion induces DNA damage and cell cycle arrest. **A** Left, NPRL2 depletion led to accumulation of a DNA damage marker, cell cycle inhibitors, and an apoptosis marker. Ras-AI-T2 cells were transduced with control or NPRL2-targeting shRNA-containing virions (20 μL virion-containing medium/10^4^ cells) for 48, 72 and 96 h. Then, cell lysates were used for immunoblot analyses. Right, paramycin might decrease NPRL2 depletion-induced cell cycle arrest and apoptosis. Ras-AI-T2 cells were transduced as described above and treated with 20 nM rapamycin (Rap.). The cells were then subjected to immunoblotting analyses. **B** NPRL2 depletion increased the percentages of cells in G1 and G2/M phases. Upper, After transduction for 48, 72 or 96 h, Ras-AI-T2 cells were collected and fixed for cell cycle analysis (lower). Alternatively, the transduced cells were treated with or without 20 nM rapamycin (Rap.) for 48 or 96 h and then collected for analysis of cell cycle distribution (lower). Results are the mean ± SD determined from three experiments. *p < 0.05 by Student’s *t*-test

**Fig. 5:**
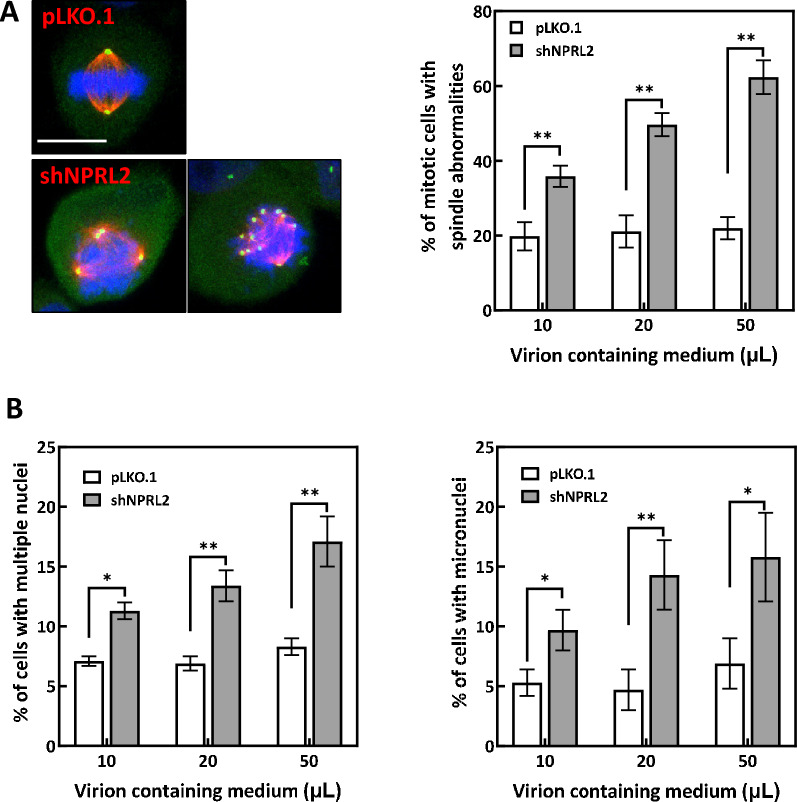
NPRL2 depletion may induce mitotic catastrophe. **A** Left, representative images of the mitotic spindles in cells with or without NPRL2 depletion. Seventy-two hours after transduction, Ras-AI-T2 cells were fixed and immunostained with anti-γ-tubulin (green) and anti-α-tubulin (red). Nuclei were counterstained with DAPI (blue). Scale bar, 10 μm. Right, the percentages of cells displaying abnormal mitotic spindles. Results are the mean ± SD from at least 200 cells for each condition, as determined from three experiments. **p < 0.01 by Student’s *t*-test. **B** NPRL2 depletion increases the percentage of cells with multiple nuclei or micronuclei. Ras-AI-T2 cells that were transduced with control shRNA virions (pLKO.1) or depleted of NPRL2 (shNPRL2) for 96 h were used for detection and quantification of multiple nuclei (left) or micronuclei (right). The percentages (mean ± SD) of cells displaying multiple nuclei or micronuclei were determined using at least 500 cells from three independent experiments. *p < 0.05 and **p < 0.01 by Student’s *t*-test

To understand how NPRL2 depletion might hamper cell proliferation, Ras-AI-T2 cells depleted of NPRL2 were collected and analyzed for phospho-Ser138 histone H2AX (γH2AX; DNA damage marker), p21 Cip1 and p27 Kip1 (inhibitors of cell cycle progression), and cleaved PARP (cPARP; apoptosis marker) by immunoblotting. The results showed that after transduction for 48 h, γH2AX and the two inhibitors of cell cycle progression were significantly increased in NPRL2-depleted cells (Fig. 4A, left). After transduction for 72 h, cPARP was also significantly accumulated in NPRL2-depleted Ras-AI-T2 cell cultures (Fig. 4A, left). Addition of rapamycin, the inhibitor of mTOR, to the NPRL2-depleted cells could significantly reduce the accumulation of p27 Kip1, p21 Cip, and cPARP, indicating the involvement of mTOR in NPRL2 depletion-induced events. Cell cycle analysis revealed that NPRL2 depletion increased the proportions of G1 cells after 24–48 h shRNA transduction, and it increased the proportion of G2 cells after 72 h shRNA transduction, as compared to controls (Fig. 4B, upper). Addition of rapamycin to the NPRL2-depleted cells could reverse NPRL2 depletion-induced cell cycle arrest (Fig. 4B, lower). These results suggest that NPRL2 depletion may induce DNA damage, block cell cycle progression, and promote apoptotic cell death, and mTOR may be involved in these events. In addition, after transduction for 72 h, the NPRL2-depleted Ras-AI-T2 cells displayed multipolar or disorganized mitotic spindles when entering into mitotic stage (Fig. 5A). After transduction for 96 h, increased numbers of the NPRL2-depleted Ras-AI-T2 cells had multiple nuclei (Fig. 5B left) or micronuclei (Fig. 5B right), both of which are features of mitotic catastrophe. Based on these findings, we conclude that NPRL2 depletion in Ras-AI-T2 cells may inhibit cell proliferation by inducing DNA damage, cell cycle arrest and apoptotic cell death, which is associated with the induction of mitotic catastrophe.

### NPRL2 depletion diminishes heat shock and antioxidant responses in oncogenic HRas-transformed cells

**Fig. 6:**
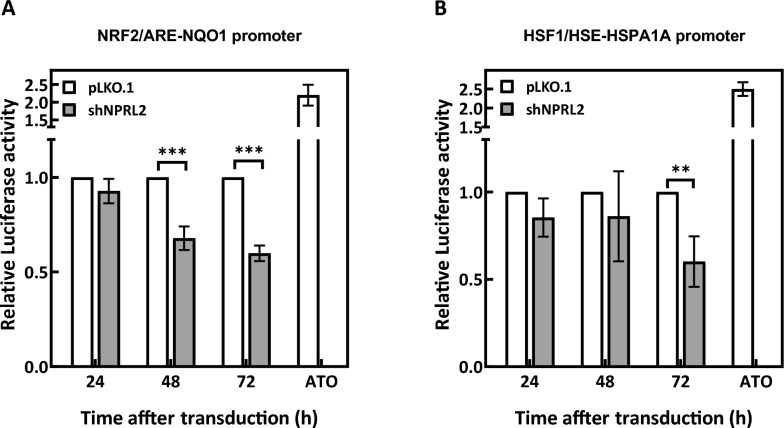
NPRL2 depletion suppresses HSE- and ARE-directed luciferase reporter activities. Ras-AI-T2 cells that were stably transfected with the NRF2/ARE- (**A**) or HSF1/HSE- (**B**) luciferase reporters were transduced with control shRNA virions (pLKO.1) or depleted of NPRL2 (shNPRL2). At the indicated times, cells were collected and assayed for luciferase activities. Treatment of the control virion-transduced cells with ATO (5 μM for 6 h) was used as a positive control for both reporters. Results are mean ± SD as determined from three independent experiments. **p < 0.01 and ***p < 0.001 by Student’s *t*-test

Oncogenic Ras-transformed cells are known to exhibit elevated levels of reactive oxygen species [31] and proteotoxic stress [32, 33]. In order to survive the oxidative damage and proteomic instability, the cells need to maintain sufficient levels of antioxidants and activate stress response machineries. We thus examined whether NPRL2 depletion might affect the antioxidant response and the heat shock response by monitoring the activities of luciferase reporters under the control of the NRF2/ARE from NQO1 promoter and HSF1/HSE from HSPA1A promoter. ATO treatment (5 μM for 6 h) of the control virion-transduced cells served as a positive control for both reporters. The results showed that both the NRF2/ARE- (Fig. 6A) and HSF1/HSE-driven (Fig. 6B) luciferase activities were significantly reduced in Ras-AI-T2 cells depleted of NPRL2 compared to those receiving pLKO.1 control. Therefore, NPRL2 depletion appears to disrupt the cellular stress response pathways mediated by NRF2, a key transcription factor for counteracting oxidative stress [34], and HSF1, the master regulator of heat shock response [35].

## Discussion

mTORC hyperactivation frequently occurs in human tumors, suggesting it may be a valuable therapeutic target. However, mTOR inhibitors have performed poorly in clinical trials, with some even displaying the cytoprotective effects of reducing oxygen and glucose consumption [21]. Thus, mTOR activity levels appears to be carefully regulated in cancer cells such that the cell can maintain the high energy demands of cell growth/proliferation without causing cell death. It would be useful to understand how the mTOR signaling pathway functions in malignantly transformed cells. In the present study, we show that depletion of NPRL2, a negative regulator of mTORC1, inhibits the proliferation of the oncogenic HRas-transformed bronchial epithelial cells in vitro and in vivo, possibly by preventing mTOR persistent activation and inducing cell cycle arrest, mitotic catastrophe and cell death. Our findings indicate that NPRL2 is required for proliferation of oncogenic HRas-transformed cells and further imply that NPRL2 may function in Ras-transformed cells to restrict excessive mTORC1 activity and allow for cell survival and transformation.

NPRL2 dysregulation has been implicated in many human pathologies, including cancers. Nevertheless, its role in cancer development is controversial. For example, studies on the expression levels and roles of NPRL2 in lung tumors have produced unclear or conflicting results. Some studies have shown strong suppression of NPRL2 gene expression and promoter hypermethylation in lung tumors [36, 37]. It was also shown that transgenic NPRL2 expression suppresses lung cancer cell proliferation in vitro by inducing apoptosis and cell cycle arrest in vitro, and it attenuates tumor xenograft formation in mice [9]. In addition, NPRL2 gene expression level is inversely correlated with cisplatin sensitivity in lung cancer cell lines [10], and NPRL2 overexpression inhibits cell growth and enhances sensitivity to cisplatin in lung cancer cell lines [10, 38]. However, NPRL2 gene expression levels are unchanged or only have small changes in most lung tumor tissues studied [12, 39]. Epigenetic analyses have also failed to show NPRL2 gene promoter hypermethylation in lung tumor tissues [12]. Furthermore, some reports have shown that NPRL2 gene or protein expression is high in some lung cancer tissues or cell lines [10, 12, 37]. Together, these studies suggest that NPRL2 may play distinct roles in different histopathological subtypes, tumor stages, or patients with different characteristics. Thus, it is critical to understand the complex mechanisms by which NPRL2 might affect lung cancer development.

Accumulated evidence indicates that mTORC1 hyperactivation drives cellular senescence and/or quiescence, as inhibition of mTORC1 can delay or even reverse the onset of senescence [40, 41]. Our results show that depletion of NPRL2 activates mTORC1 downstream signaling and inhibits the proliferation of Ras-transformed cells, induces dramatic increases in the levels of γH2AX, upregulates cell cycle inhibitors p21 and p27, increases the proportions of G1 and G2 cells, and induces mitotic catastrophe. Therefore, cells with downregulated NPRL2 and activated mTORC1 might be expected to enter into a senescent (or quiescent) state, and we can conclude that NPRL2 is required for survival and proliferation of the transformed cells. In addition, our results showed that rapamycin, a mTORC1 inhibitor, could rescue NPRL2 depletion-induced cell death, indicating NPRL2 depletion-induced mTORC1 activation may cause cell death in oncogenic HRas-transformed cells. These conclusions are consistent with a previous report showing that NPRL2 expression is upregulated in prostate cancers and linked to poor prognosis [13] and another showing that NPRL2 depletion inhibits proliferation of prostate cancer cells [14]. In addition, NPRL2 was found to be significantly upregulated in docetaxel-resistant prostate cancer cells and associated with decreased mTOR signaling and elevated autophagy; depleting NPRL2 in these cells increases sensitivity to docetaxel by inducing apoptosis [16]. Our results also revealed that NPRL2 depletion activated mTOR downstream signaling, blocked autophagy, induced cell cycle arrest and apoptosis. Along with these studies on prostate cancer, our study supports the idea that NPRL2 is required for cancer cell survival and proliferation in some contexts.

Modulation of mTOR activity has been studied in other contexts as well. For example, chronic activation of mTORC1 in NPRL2 knockout skeletal cells leads to compensatory increases in anaplerotic pathways to replenish TCA intermediates that are consumed for biosynthetic purposes [27]. Those results suggest that persistent mTORC1 hyperactivation may disrupt the homeostatic regulation of mitochondrial functions. Along these lines, primary human fibroblasts with hyperactivated mTOR are known to be deficient in mitophagy, which leads to accumulation of damaged mitochondria and consequent increases in oxidative stress [42]. Furthermore, mTORC1 activation in NPRL2 knockout murine embryonic fibroblasts causes impairments in amino acid metabolism and homeostasis [43]. Similarly, murine embryonic fibroblasts with deficiencies in tuberous sclerosis complex (TSC; negative regulator of mTOR) have hyperactivated mTOR and are more susceptible to necrotic cell death [44]. Another report showed that deficiency of TSC in intestinal epithelial cells induces TUNEL-positive cell death [45]. These studies revealed that NPRL2 depletion and chronic activation of mTORC1 may disrupt cellular homeostasis and impair cell proliferation. Our results also showed that NPRL2 depletion-induced cell death could be rescued by inhibition of mTOR with rapamycin in oncogenic Ras-transformed cells. Thus, NPRL2 inhibition of mTORC1 in Ras-transformed cells may paly critical roles to support cell survival and proliferation.

Our experiments showed that NPRL2 depletion in Ras-AI-T2 cells reduced NRF2- and HSF1-directed luciferase reporter activities, suggesting that the antioxidant response and heat shock response were suppressed. These two cytoprotective processes allow cells to cope with stresses that might arise from excessive proliferation, as would occur in oncogenic Ras-transformed cells. Previous studies have shown that the mitogenic response of oncogenic Ras involves the production of reactive oxygen species [46–48]. By damaging cellular macromolecules, excessive levels of reactive oxygen species are highly detrimental to the cells, potentially causing cell cycle arrest, senescence or cell death [49]. To combat high levels of reactive oxygen species, the NRF2-mediated antioxidant response is upregulated in oncogenic Ras-transformed cells, and its disruption will impair cell proliferation and tumorigenesis [50]. Similarly, HSF1 can be activated by oncogenic Ras [51] and is required for oncogenic Ras-induced malignant cell transformation [32]. Thus, it is reasonable to expect that HSF1-mediated heat shock response may protect the transformed cells from excessive proliferation-induced proteotoxic stress inherent to malignant transformation. These studies imply that suppression of the NRF2-mediated antioxidant response and HSF1-mediated heat shock response by NPRL2 depletion may disrupt the cytoprotective mechanisms and allow damage to accumulate in the Ras-transformed cells, thus hampering the survival and proliferation of Ras-AI-T2 cells. In addition, mTORC1 has been shown to sense and negatively respond to oxidative and proteotoxic stresses as an adaptive strategy to support cell survival under stress conditions [52, 53]. In our study, NPRL2 depletion in Ras-AI-T2 cells may induce persistent activation of mTORC1, which may worsen cell sensitivity to oxidative stress and proteotoxicity that might be overly produced in the transformed cells.

In summary, our study demonstrates that NPRL2 is required for survival and proliferation of oncogenic HRas-transduced and malignantly transformed bronchial epithelial cells. Furthermore, our data imply that the malignantly transformed cells may depend on NPRL2 to restrict excessive mTORC1 activity, thereby promoting tumorigenic cell survival and proliferation.

## Materials and methods

### RNA-sequencing data acquisition from The Cancer Genome Atlas (TCGA) and Genotype-Tissue Expression (GTEx) databases

The NPRL2 gene expression data from TCGA and GTEx-normal were retrieved using the UCSC Xena platform [25]. As these human data are from public online databases, an institutional approval is not required for the use of these data. RNA-sequencing data from 1013 primary and recurrent lung tumors and 109 adjacent normal lung tissues were retrieved from TCGA-lung cancers, while data from 288 normal lung tissues were obtained from GTEx. RNA-sequencing data from 496 primary and recurrent prostate tumors and 52 adjacent normal prostate tissues were also retrieved from TCGA-prostate cancers, while data from 100 normal prostate tissues were collected from GTEx.

### Cell culture and chemicals

BEAS-2B cells were obtained from the American Type Culture Collection (ATCC, Manassas, USA) and maintained in Dulbecco’s Modified Eagle’s medium (DMEM, Gibco, Thermo Fisher Scientific, MA, USA) with 10% (v/v) fetal bovine serum (Gibco), 100 mg/mL penicillin and 100 mg/mL streptomycin in an incubator with a fully humidified 10% CO_2_ atmosphere at 37 °C. The cells containing empty vector pFB-Neo or stably expressing FLAG-tagged HRas^G12V^ were established as described [54]. Ras-AI-T2, a HRas^G12V^-transduced cell clone was considered to be malignantly transformed due to its abilities to undergo anchorage-independent growth and xenografted tumor formation. The effects of NPRL2 depletion on cell proliferation of Ras-AI-T2 (oncogenic HRas-transduced and malignantly transformed bronchial epithelial) cells were assessed in culture by trypan blue exclusion and annexin V binding apoptosis assays, colony formation assays, and soft agar colony formation assay. Cell proliferation was also evaluated in a xenograft mouse model.

### Depletion of NPRL2 and qPCR

shRNAs targeting NPRL2 (gene symbol TUSC4, TRCN-38079 and 38083) were purchased from the National RNAi Core Facility (Genomic Research Center, Academia Sinica). The shRNA-containing virions were prepared, and depletion of endogenous NPRL2 was performed as described previously [55]. Empty vector pLKO.1-containing virions were also prepared and used as a control. Forty-eight hours after transduction, total RNA or cell lysates were collected, and the expression of NPRL2 gene and proteins were assessed by qPCR and immunoblotting. Total RNA was extracted from cells with Trizol (Thermo Fisher Scientific) following the manufacturer’s protocol. A 2.5 μg aliquot of total RNA was reverse-transcribed into cDNA with the PrimeScriptII RT Enzyme kit (Takara Bio USA). Then, qPCR was performed with a Light Cycler 480 system (Roche, Penzberg, Germany). The amplification reaction was performed in a 20 μL mixture containing 10 μL 2 × SYBR Green mix, 1 μL cDNA, and 0.2 μM of each primer. The PCR conditions were as follows: denaturation at 95 °C for 5 min; 40 cycles of 95 °C for 10 s, 60 °C for 10 s, and 72 °C for 10 s. Following amplification, the PCR products were examined via melting curve analyses. NPRL2 RNA levels were normalized to GAPDH mRNA, and each sample was analyzed in triplicate. mRNA levels were assessed by calculating 2^−ΔΔCt^ values. The primer sets used were as follows: NPRL2, forward 5′-TGCACTCTGCCCATTGATGA-3′ and reverse 5′-AGGTACATCATACTCCTGGG; GAPDH, forward 5′-CTCCTCCACCTTTGACG-3′ and reverse 5′-ACCACCCTGTTGCTGTA-3′.

### Immunoblotting

Cell lysis and immunoblotting were performed as described [55]. Proteins were detected using anti-NPRL2 (GTX54626, GeneTex, Taiwan), anti-phospho-Thr389-p70 S6 Kinase (#9205, Cell Signaling Technology, MA, USA), anti-phospho-Thr37/46-4EBP1 (#2855, Cell Signaling Technology), anti-p27 Kip1 (#3686, Cell Signaling Technology), anti-p21 Cip1 (#2947, Cell Signaling Technology), anti-cleaved PARP (#5625, Cell Signaling Technology), anti-LC3B (#3868, Cell Signaling Technology), anti-SQSTM1/p62 (#5114, Cell Signaling Technology), anti-phospho-Ser139-Histone H2A.X (#9718, Cell Signaling Technology). PCNA and HSC70 served as loading controls and were detected with anti-PCNA (sc-56, Santa Cruz Biotechnology, TX, USA) and anti-HSC70 (sc-7298, Santa Cruz Biotechnology).

### Cell proliferation and apoptosis assays

To evaluate the proliferative capacity of cells depleted of NPRL2, Ras-AI-T2 cells were transduced with control or NPRL2 shRNA-containing virions (10 or 20 μL virion-containing medium/10^4^ cells). After 48 h, cells were replated, and the viability and proliferation rate were tracked by performing a trypan blue exclusion cell counting assay every 24 h for 4 days [56]. Apoptosis was detected as previously described [57] with annexin V-FITC/PI staining kit (BD Bioscience, New Jersey, USA).

### Colony formation assay

Cells were transduced as described above. After 48 h, cells were trypsinized, counted, replated into 60-mm petri dishes, and cultured for 10 days to allow colony formation [58]. The assay was performed in triplicate for each sample. The colonies were then fixed with methanol, stained with 1% Giemsa, air-dried, and counted. Plating efficiency (PE) was calculated as the ratio of the number of colonies to the number of cells seeded; cells transduced with the control pLKO.1-containing virions were taken as 100%.

### Soft agar colony formation assay

The virion transduction and cell replating procedures were the same as described for the colony formation assay, except that cells were first suspended in 0.3% agarose-containing medium and then spread onto an 0.6% agarose-containing medium underlay [59]. The assay was performed in triplicate for each sample. Every 7 days, approximately 200 μL of fresh media was added dropwise to each dish in order to keep the agar hydrated. The single cells were allowed to form colonies for 21 days. Colonies were fixed, stained and counted. Colonies were scored using a visual counting grid under a dissecting microscope. Relative colony number for each soft agar plate was obtained by normalizing the colony number in the plate to the PE in a solid plate.

### Cell growth in xenograft mouse model

The design and study procedures were approved by the Institutional Animal Care and Use Committee of Academia Sinica (Protocol ID: 12-12-436). Male athymic nude mice (4–5 weeks old, with a bodyweight of 18–20 g) were obtained from the National Laboratory Animal Center (Taipei, Taiwan) and housed for 1 week in a pathogen-free animal facility before experimental manipulation. Ras-AI-T2 cells were transduced with virions containing empty vector (pLKO.1) or shRNA targeting NPRL2 (shNPRL2) at a density of either 10 or 20 μL virion-containing medium/10^4^ cells. After transduction for 48 h, the cells were collected, suspended in PBS (pH 7.4), and then immediately subjected to implantation. For implantations, an aliquot of Ras-AI-T2 cells (2 × 10^6^ cells) suspended in 100 μL PBS was subcutaneously inoculated into the hind limb of a mouse. To monitor tumor growth, the longest and shortest diameters of the tumors were measured using calipers. Tumor volume (mm^3^) was calculated according to the following formula: tumor volume = (length × width^2^)/2. Mouse body weight was also measured as a general indicator of health.

### Immunofluorescence staining and analysis of autophagy and spindle abnormalities

Cells were seeded on coverslips and then transduced with control or NPRL2-targeting shRNA-containing virions. After 72 h, the cells were fixed and subjected to immunofluorescence staining as previously described [55]. The primary antibodies included anti-LC3B (#3868, Cell Signaling Technology), anti-LAMP1 (#9091, Cell Signaling Technology), anti-α-tubulin (GeneTex or Sigma) and anti-γ-tubulin (Sigma). Alexa-Fluor 488- and 633-conjugated goat anti-mouse and anti-rabbit IgG were purchased from Invitrogen (Carlsbad, CA, USA). For analysis of autophagy abnormalities, the autophagosomes were immunostained for LC3 and LAMP1, as described above. Confocal imaging of the immunostained samples was performed using a Leica TCS-SP5 upright microscope. For each sample, the percentage of cells containing puncta were positively stained for LC3 but not LAMP1 was calculated. For analysis of mitotic spindle abnormalities, the mitotic spindle was revealed by immunostaining for α- and γ-tubulins, as described above. The numbers of cells with mitotic spindle abnormalities were counted under a Zeiss Axioplan 2 Imaging MOT fluorescence microscope. Multipolar spindles and disorganized spindles were regarded as mitotic spindle abnormalities. The level of spindle abnormalities is expressed as the percentage of the mitotic cells containing abnormal spindles.

### Analysis of cell cycle distribution

Cell cycle progression was monitored using DNA flow cytometry as described [60]. DNA was stained with propidium iodide (PI), and the DNA contents of individual cells were analyzed using a fluorescence activated cell sorter (Attune NxT, Thermo Fisher Scientific, Invitrogen). The cell cycle distributions were determined using a computer program provided by Invitrogen.

### Analysis of cells with multiple nuclei and micronuclei

Cells (1 × 10^4^) were seeded on coverslips and transduced with the indicated volume of medium containing control or NPRL2-targeting shRNA virions. After 96 h, the cells were fixed with methanol and stained with 3% Giemsa (BDH, Bristol, UK). The coverslips were mounted, and cells were examined under an under a Zeiss Axioplan 2 microscope. Cells with multiple nuclei or micronuclei were identified and counted.

### Luciferase reporter assays

Two luciferase reporter cell lines were established by G418 selection of Ras-AI-T2 cells stably expressing firefly luciferase reporter genes under the control of different promoters. HSPA1A-Luc was driven by the heat shock factor 1 binding heat shock element (HSF1/HSE) of HSPA1A [54], and NQO1-Luc was driven by the NRF2/antioxidant response element (ARE) of NAD(P)H quinone oxidoreductase 1 (NQO1) [61]. After transduction with control virion or virion containing shRNA targeting NPRL2 (20 μL virion-containing medium/10^4^ cells) for the indicated times, the luciferase reporter cells were collected and luciferase assays were performed according to the manufacturer’s instruction (Bright-Glo assay system, Promega). Luciferase activities were normalized to the protein amount applied in each assay. Treatment of control virion-transduced cells with 5 μM arsenic trioxide (ATO) for 6 h was used as a positive control for both reporters.

### Statistics

All statistical analyses are described in the figure legend and were performed using GraphPad Prism 9.12 software.

## Data Availability

The data generated or analyzed during this study are contained in this published article.
